# Medical College Convention

**Published:** 1870-06

**Authors:** 


### MEDICAL COLLEGE CONVENTION.

The Delegates from Medical Colleges assembled in the Lecture-room of the Georgetown Medical College, corner of 10th and E. Streets, Washington, D.C., on the morning of April 20th, 1870; and were called to order at 11½ oclock A.M., by Prof. N. S. Davis, of Chicago. Prof. S. D. Gross was elected temporary Chairman, and N. S. Davis Secretary.

On motion of Prof. S. M. Bemis, the Chair was requested to appoint a Committee of three to verify the credentials and make a list of members. The Chair appointed Professors S. M. Bemis, J. S. Moore, and A. Stille such Committee.

Prof. Bemis, in behalf of the Committee, reported the following list of Delegates, with the names of the Institutions they represent, *viz.*:—

| Institutions Represented. | | Names of Delegates. |
|---|---|---|
| N. O. School of Medicine, | Prof. | Sam'l Logan. |
| Howard University of D.C., | " | Robert Reyburn. |
| " " " | " | Silas L. Loomis. |
| University of South Carolina, | " | A. N. Talley. |
| " " " | " | John T. Darby. |
| Detroit Medical College, | " | E. W. Jenks. |
| Missouri Medical College, | " | J. S. Moore. |
| Chicago Medical College, | " | N. S. Davis. |
| Med. Depart. Georgetown Col'ge, | " | J. H. Thompson. |
| " " " " | " | C. C. Cox. |
| Med. Depart. Williamette Uni'ty, | " | Horace Carpenter. |
| University of Louisiana, | " | S. M. Bemis. |
| Jefferson Medical College, | " | S. D. Gross. |
| University of Pennsylvania, | " | F. G. Smith. |
| " " " | " | Alfred Stille. |
| St. Louis Medical College, | " | J. B. Johnson. |
| Wash. University, Baltimore, Md., | " | Chas. W. Chancellor. |
| University of Louisville, | " | David W. Yandell. |

On motion, the report of the Committee was accepted. A motion was then adopted, requesting the Chairman to appoint a Committee of five to nominate permanent officers of the Convention; and Professors C. C. Cox, F. G. Smith, D. W. Yandell, Sam'l Logan, and N. S. Davis were appointed such Committee.

The Convention then took a recess of fifteen minutes, to allow the Committee time for consultation. When the Convention was again called to order, Prof. Cox, Chairman of Committee on Nomination, responded as follows: President, Prof. S. D. Gross, of the Jefferson Medical College, Philadelphia; Vice-President, Prof. David W. Yandell, of the University of Louisville, Ky.; Secretary, Prof. N. S. Davis, of the Chicago Medical College, Ill.

On motion, the report of the Committee was accepted, and the nominations unanimously confirmed. Prof. Gross cordially thanked the Convention for the honor conferred on him, and expressed a deep interest in the accomplishment of the objects for which the Convention had assembled.

The proceedings of the previous Convention, held in Cincinnati, May 3d, 1867, being called for, Prof. Davis, Chairman of the Committee appointed by that Convention, presented and read a report, stating fully the result of the deliberations of the previous Convention, and the subsequent action of the Colleges in relation thereto, as follows:—

The first attempt to procure concert of action in the work of improving the system of Medical College instruction in this country, by a Convention of Delegates from Colleges alone, was made on Monday, preceding the meeting of the American Medical Association, in Louisville, in May, 1859, and was followed by a similar meeting, the day preceding the assembling of the Association, in New Haven, in June, 1860. Less than a majority of the Medical Colleges in the country were represented in both of those meetings, and no attempt was made to effect any considerable changes in the methods of instruction. Circumstances beyond the control of the medical profession, prevented any further action in this direction, until the American Medical Association, during its session in Baltimore, in May, 1866, adopted a resolution, earnestly requesting the Medical Colleges of the whole country to hold a Convention of Delegates from their own faculties, and thoroughly revise our whole system of medical college instruction; and appointed a Committee to aid in carrying the resolution into effect. It was in response to a circular issued by that Committee that a Convention of Delegates from Medical Colleges assembled in Cincinnati, May 3d, 1867. Delegates were present from eighteen Medical Colleges, constituting little more that half the number then existing, and in active operation in the United States, and including several of the oldest and most influential character. Prof. A. Stille, of the University of Pennsylvania, presided, and Prof. G. C. E. Weber, of Charity Hospital Medical College, acted as Secretary. After three days spent in candid and full discussion of the whole subject, the following propositions were adopted by nearly an unanimous vote, namely:

(*See Circular Letter on next page.*)

Having thus agreed upon a thorough revision of our whole system of Medical College instruction, the Convention adopted the following resolution, *viz.:*

*Resolved,* That a Committee of five be appointed by the President, whose duty it shall be to present the several propositions adopted by the Convention to the Trustees and Faculties of all the Medical Colleges in this country, and solicit their definite action thereon, with a view to the early and simultaneous practical adoption of the same throughout the whole country. And that the same Committee be authorized to call another Convention, whenever deemed advisable.

In obedience to this resolution, the President appointed N. S. Davis, of Chicago; S. D. Gross, of Philadelphia; F. Donaldson, of Baltimore; Alden Marsh, of Albany; and Geo. C. Blackman, of Cincinnati, to constitute said Committee.

As soon after the adjournment of the Convention as practicable, the Committee, in obedience to the foregoing resolution, prepared the following circular letter, and sent copies of the same to the Trustees and Faculties of every regular Medical

College in the United States, the existence of which was known to the Committee at that time. The letter is as follows:

TO MEDICAL COLLEGES.

At the Convention of Delegates from Medical Colleges, called for the purpose of revising the system of Medical College instruction in this country, and which convened in Cincinnati, May 3d, 1870, the following resolution was unanimously adopted:—

"RESOLVED, That a committee of five be appointed by the President, whose duty it shall be to present the several propositions adopted by this Convention, to the Trustees and Faculties of all the Medical Colleges in this country, and solicit their definite action thereon, with a view to the early and simultaneous practical adoption of the same throughout the whole country. And that the same committee be authorized to call another convention whenever deemed advisable."

The undersigned Committee, appointed for the purpose of carrying into effect the instructions contained in the foregoing resolution, respectfully invite the attention of the Trustees and Faculty of ———— to the five following propositions, which, after mature deliberation, were adopted by the said Convention with entire unanimity:—

"RESOLVED, 1st. That every student applying for matriculation in a Medical College shall be required to show, either by satisfactory certificate, or by direct examination by a committee of the Faculty, that he possesses a knowledge of the common English branches of education, including the first series of mathematics, the elements of the natural sciences, and a sufficient knowledge of Latin and Greek to understand the technical terms of the profession: and that the certificate presented, or the result of the examination thus required, be regularly filed as a part of the records of each Medical College.

2d. That every medical student shall be required to study four full years, including three regular annual courses of Medical College instruction, before being admitted to an examination for the degree of Doctor of Medicine.

3d. That the minimum duration of a regular annual lecture term, or course of Medical College instruction, shall be six calendar months.

4th. That every Medical College shall embrace in its Curriculum the following branches, to be taught by not less than nine Professors, viz.:—Descriptive Anatomy, including dissections; Physioloy and Histology; Inorganic Chemistry; Materia Medica; Organic Chemistry and Toxicology; General Pathology, Therapeutics, Pathological Anatomy, and Public Hygiene; Surgical Anatomy and operations of Surgery; Medical Jurisprudence and Medical Ethics; Practice of Medicine; Practice of Surgery; Obstetrics, and Diseases of Women and Children; Clinical Medicine and Clinical Surgery; and that these several branches shall be divided into three groups or series, corresponding with the three courses of Medical College instruction required.

The first, or Freshman series, shall embrace Descriptive Anatomy and Practical Dissections; Physiology and Histology; Inorganic Chemistry and Materia Medica. To these the attention of the student shall be mainly restricted during his first course of Medical College instruction, and in these he shall submit to a thorough examination by the proper members of the Faculty, at its close, and receive a certificate indicating the degree of his progress.

The second, or Junior series, shall embrace Organic Chemistry and Toxicology; General Pathology, Pathological Anatomy, Therapeutics, and Public Hygiene; Surgical Anatomy and operations of Surgery; Medical Jurisprudence and Medical Ethics. To these the attention of the medical student shall be directed during his second course of Medical College instruction, and in them he shall be examined at the close of his second course, in the same manner as after the first.

The third, or Senior series, shall embrace Practical Medicine; Practical Surgery; Obstetrics and Diseases peculiar to Women and Children; with Clinical Medicine and Clinical Surgery in a hospital. These shall occupy the attention of the student during his third course of College instruction, and at its close he shall be eligible to a general examination for the degree of Doctor of Medicine.

The instruction in the three series is to be given simultaneously, and to continue throughout the whole of each annual College term; each student attending the lectures on such branches as belong to his period of progress in study, in the same manner as the sophomore, junior and senior classes, each pursue their studies simultaneously throughout the Collegiate year in all our Literary Colleges.

5th. That every Medical College should immediately adopt some effectual method of ascertaining the actual attendance of students, upon its lectures and other exercises, and at the close of each session, or of the attendance of the subject, a certificate specifying the time and the courses of instruction actually attended, should be given, and such certificates only should be received by other Colleges as evidence of such attendance."

It will be seen that these propositions are designed to introduce into the system of Medical College instruction in this country, four changes of great practical importance, namely:—1st. A positive standard of preliminary education. 2d. A longer time in which to acquire a knowledge of the various branches of Medical Science and practice. 3d. A systematic and successive order of studies for the student. 4th. A certain amount of direct Clinical instruction in a public Hospital as a part of the senior course. The desirableness of these changes is too apparent to require either argument or illustration. The plan for accomplishing them, adopted by the Convention, as expressed in the foregoing propositions, is simple and easy of execution, provided the several Colleges will act in concert.

It requires each College to obtain and place on record sufficient evidence that every student admitted to matriculation possesses a certain amount of preliminary education. It requires attendance and pay for three annual courses of College instruction, as a condition for graduation; and arranges the whole curriculum of the College into three corresponding series of branches, so that each student can limit his attention to one series each year, thereby laying a foundation and building on it a superstructure in their natural order.

It contemplates such an increase in the number of members of each College Faculty, that four lectures per day can be given to each of the three classes in attendance, throughout the whole College term of six months. This would afford a very full course of instruction in each of the three series of branches, and yet give to the members of each class time fully to digest the instruction received. This would make it necessary during a part of each day that lectures should be given at the same hours to different classes. But as all Medical Colleges contain two, and some of them three Lecture rooms, this would be attended by no inconvenience to the Faculty or students. The only valid objection which has been suggested by those connected with the Medical Colleges is, that the increase in the number of each College Faculty required by the proposed plan, would necessitate a corresponding greater division in the income of each College, and thereby seriously reduce the amount received by any one member. If it is remembered, however, that while the plan requires a moderate addition to the number of members in the Faculties of most of the Colleges, it also requires each student to attend and pay full fees for three courses of instruction instead of two, it will be seen that the revenues of each College derived from Lecture fees, will be increased in full proportion to the increase of the Faculty. As most of the Colleges have allowed each member of the Faculty to sell his ticket to the class and retain the proceeds as his individual compensation, it has been thought that the proposed division of the students, attending any given College, into three distinct classes, and assigning to each a distinct series of branches, would limit the sale of the tickets of any one Professor to the special class receiving instruction in his department; and consequently would restrict his income in proportion to the restricted number of tickets sold.

This is simply a misapprehension. The Convention took no action regarding the rate of lecture fees in any of the Colleges; but the plan proposed was founded on the expectation that each student would pay the same aggregate fees annually, as under the old plan. For instance, a student attending the first course, and taking out the four tickets of the Freshmen series, would pay the same amount for the four that he now pays for the seven or eight that cover the curriculum in most of the Colleges at this time. Hence, although each member of the Faculty would sell a smaller number of tickets, the income from them would be nearly the same. Or each College could have all lectures free, from the several divisions of the class, paid to a common treasurer, and each member of the Faculty allowed to draw on such treasurer for his proportion of the same.

The fourth section or proposition adopted by the Convention was not designed to fix the titles to Professorships in the Colleges, but simply to designate what was deemed necessary to constitute a proper Medical College Curriculum, and to determine what part of the Curriculum should be included in each of the three series of studies. Uniformity among the Colleges in regard to this division into series, is very desirable, in order to enable students, if they choose, to attend one series of studies in one College and another series in another, without confusion.

To obviate embarrassment in making the change from the present system of College instruction to the one proposed, we would suggest that all students who should have so nearly completed their period of study at the time fixed for making the change, than an attendance on a single additional course of Lectures would render them eligible to graduation, should be allowed to complete their course by attending the senior department under the new arrangement; while all who are in the first half of their period of study, should be subject to the new arrangement in full.

That the interests of medical science, the honor of the profession, and the welfare of the people, urgently require important improvements in our system of medical education and Medical College instruction, is apparent to all. The public sentiment of the profession, as expressed through the National, State, and local Societies, and through the leading medical periodicals, cordially sanctions the plan here proposed. We, therefore, respectfully ask you to give it a full consideration, and return to the Chairman of the undersigned committee answers to the following questions:—

1st. Do your Faculty, together with the governing authority of your College, approve of the several propositions as a whole?

2d. If you do not approve of the plan of revision as a whole, what changes would you suggest?

3d. If you approve of the plan as a whole, or of all its essential features, will your College be ready to adopt it practically, and issue your annual announcement for the College term of 1868–9, in accordance therewith; provided all the principal Medical Colleges in this country (or, at least, those in the cities of Boston, New York, Philadelphia, Baltimore, Richmond, Charleston, New Orleans, Louisville, Cincinnati, St. Louis, Chicago, Buffalo, and Albany) will agree to do the same at the same time?

The great desideratum is to secure both harmony and concert of action on the part of the Medical Colleges, in the adoption of such measures as will at once place the system of medical education in this country on such a basis as the extent of the science and the responsibilities of its practical application in the prevention and treatment of diseases require.

N. S. Davis,<br>

S. D. Gross,<br>

Geo. C. Blackman,<br>

F. Donaldson, } Committee.

Chicago, August 1, 1867.

To remedy the effects of having accidently omitted any Schools, the same letter was published in most of the medical periodicals at the same time. To the circular letter thus addressed and its plain questions, official answers were received from the Faculties of only ten Medical Colleges, while the number then in active operation was over thirty.

The ten replies mentioned were from the Medical Department of Harvard University; the Medical Department of Yale College; the Jefferson Medical College; Medical Department University of Michigan; Miami Medical College, Cincinnati; Medical College of the State of South Carolina, Charleston; Humboldt Medical College, St. Louis; Talland Medical College, San Francisco; Med. Depart. of Williamette University, Salem, Oregon; and the Chicago Medical College, now Medical Depart. of the North-Western University. As none of these replies are lengthy, we submit them in full, in the order mentioned.

BOSTON, *Sept. 23, 1867.*

MY DEAR SIR:—The circular to Medical Colleges, addressed by the Committee, was received and submitted to the Faculty. It was discussed pretty freely, but as the propositions cannot be adopted and carried out now as a whole, it was voted to postpone the further consideration of the subject. With regard, truly yours, GEO. C. SHATTUCK.

---

MEDICAL INSTITUTION OF YALE COLLEGE.
NEW HAVEN, *April 6, 1868.*

N. S. DAVIS, M.D., *Chairman of Committee of Med. Teachers' Convention.*

*Dear Sir:*—The circular issued by your Committee, presenting to the Faculty of the College the several propositions adopted by the Convention of Medical Teachers held last year, and soliciting definite action thereon, to the end that they may be simultaneously and practically adopted throughout the whole country, has been fully considered by the Faculty; and the undersigned have been directed to transmit to your Committee the following reply:

In doing so, however, we cannot neglect this opportunity of expressing, both for our colleagues and ourselves, our active sympathy with every well-considered movement, having for its object the improvement of the profession; and particularly the elevation of the standard of education, both general and professional, which is necessary to secure admission to its ranks; and our high appreciation of the efforts of the gentlemen engaged in the recently renewed attempt to attain that object—for it is a work that this College, ever since its formation, has been actually engaged in. The relations which this Institution sustain to the Connecticut Medical Society, by which it was originated, and to the State Legislature, by which it was chartered, and made one of the Departments of Yale College are such, that any radical change in its plans of instruction cannot be suddenly or easily effected. This is not the first occasion on which this College has been solicited to change entirely its *curriculum* of study, as well as the prerequisites for graduation.

More than forty years ago, as doubtless your Committee are aware, the Connecticut Medical Society sent representatives to a Convention of Delegates, from Medical Societies and Colleges, held at Northampton, pursuant to a call issued by the Medical Society of Vermont, for the purpose of devising plans for elevating the character of medical education.

After discussing the various subjects which had been suggested by the circular of the Vermont Society, and such as were proposed by members of the Convention, certain regulations (twelve in number) were adopted, together with by-laws and resolves, providing for making known to the several Medical Colleges and Societies of the United States the results of their deliberations,—offer their ratification of them.

As your Committee are doubtless familiar with the proceedings of the Convention, it is unnecessary to do more than to allude to the very clear and forcible presentation of the arguments by which their propositions were enforced, or to enter into a detailed statement of the changes proposed, further than to mention that "each candidate for a license to practice, or for the degree of Doctor of Medicine, was required to present satisfactory evidence that he had received from some respectable College the degree of Bachelor of Arts; or that *previous to the commencement* of his professional studies he had acquired a good English education; and such knowledge of the Latin language as to enable him to read with facility the Æneid of Virgil and the select Orations of Cicero; and that he had also obtained a good acquaintance with the principles of Geometry and Natural Philosophy." Such students as were regular graduates of Colleges, were required to study for *three* years, and attend two courses of public lectures—those who were not graduates of Colleges, were required to study *four* years.

The records of the Connecticut Medical Society show that it adopted unanimously the recommendations of the Convention; and entering at once and heartily into the movement, to advance the standard of medical education, it procured, at some trouble, the necessary alterations of the laws of the State, and the charter of the Medical College, so as to conform them to the new system. The Faculty earnestly seconded these efforts, and their action was made to conform to the standard demanded by the Northampton Convention, in 1827.

The new system was faithfully pursued by this College for three consecutive years, and until it became evident that the solemn compact was no longer regarded by the other Medical Colleges represented in the Convention, or who had subsequently signed the agreement. One after another they refused to be bound by their own regulations, until finally this Institution, according to the traditions of the fathers, found itself standing alone—"faithful among the faithless,"—in the combined effort to advance the true interests of medical education in the United States.

Seeing that the students were attracted away to other institutions, which practically ignored the new regulations to which they had all pledged themselves, and finding that a longer perseverance in the extended course of instruction would not only injure the Institution, but would fail to be of any benefit to the cause of medical education, the Connecticut Medical Society, in 1832, voted "that the Committee on the term of study report to the General Assembly a bill, in due form, for a public Act, to alter the term of study, making the time required the same as by the law in force previous to the Convention, in 1827." The bill, as presented, became a law the same year, and the *term of study* in this Institution has remained until the present time unchanged.

In regard to the *third* question of the circular, then, we have decided to answer in the negative.

In reply to the *second* question, we are convinced that although the propositions of the Committee are in themselves unobjectionable, they are inadequate as a remedy for the great evils of the present system of education.

The great salient evils of this system are, in our opinion,

1st. The almost total absence of *adequate preparatory education* in the young men who enroll themselves as students of medicine.

2nd. The fact that the greater portion of these receive no instruction whatever worthy of the name, nor indeed accomplish *any systematic study*, in the proper sense of that term, *before* they listen to public lectures—if they ever do.

3rd. Too great prominence is already given to public lectures, and too little to *daily text-book recitations.*

And the student who comes fresh from the plough and the work-bench, with only the preparatory education which the district school affords, or the shrewdness acquired in some business pursuit, receives his degree after the same term of study as is required of the most thoroughly disciplined graduate of a University.

Such a palpably gross evil as this requires no comment. In order to remedy, so far as its influence could reach, the *first* evil enumerated, the Connecticut Medical Society, many years ago, resolved, in substance, that hereafter no young man should be received into the office of any of its members, as a student of medicine, until he had passed a satisfactory examination, by his instructor and a Fellow of the Society, in the leading branches of English education; also in the Greek and Latin languages—in effect, very nearly the examination that was then required for admission to the Freshman Class in Yale College. This was placing the standard so far above the demands of the public—of the other Medical Colleges, and even of the profession generally, that the law soon became imperative, and so continues, although we hope it will yet be revived.

*Third.*—The system of teaching medicine by daily text-book recitations and familiar lectures, through a large part of the year, which is in operation in Yale College, in addition to the course of public lectures and Hospital facilities, we believe to be the only proper one. No student should be graduated *anywhere*, unless his studies have been faithfully and systematically pursued, under circumstances that insure the full occupation of his time, to the exclusion of all other business, and under the direction of a competent teacher. If a student thus educated can only be admitted to graduation after three years of constant application, *when* ought those to graduate, who read medicine as the majority do, without recitations of any kind, and with only nominal instruction?

It is not an extension of the lecture term to six months, nor yet a great multiplication of the subjects of study, that can diminish, much less remove the great evils that we all feel do exist. The only change that can reach them is the absolute requirement of greater preparatory knowledge; and the adoption of the mode of daily teaching in classes, in public institutions, instead of the loose and indefinite one that now so universally prevails in the offices of physicians.

As evidence, if any were needed, of our desire to advance as far as practicable, the standard of medical education, we will mention the fact already known to your Committee that we have it in contemplation, at no distant day, to perfect plans already in process of completion, by which the Medical Sciences will be taught here as the other Sciences are taught, to graded classes, by daily text-book recitations and lectures, throughout the Academic year. In a democratic country like ours, where educational interests are in no sense fostered or controlled by a central government, and where the quality of education, as of other things, is regulatəd by the public demands, the attempt to bring all Medical Colleges to adopt the same greatly advanced and prolonged course of study, and to compel all students to come up to that standard, *before the public mind is sufficiently educated to appreciate and demand it*, is in our judgment premature, and not likely to prove successful.

While for these and other reasons the Faculty deem it inexpedient to adopt at present the recommendations of the Con-Convention, they will be prepared to give them a favorable consideration, and to adopt them, so far as our circumstances will allow, *whenever they are adopted and faithfully adhered to as the uniform and settled practice of the leading Medical Colleges of this country.*

Appreciating fully your zeal and devotion in the cause of medical education, which we ourselves have so much at heart, and hoping to see the day when our ideal in this regard may be realized, we are, with great respect and esteem, respectfully yours,

S. G. HUBBARD, M.D.,<br>M. C. WHITE, M.D., } *Committee of Faculty.*

---

ST. LOUIS, MO., *April 11th, 1868.*

N. S. DAVIS, M.D., *Chairman of Committee on Med. College Instruction.*

SIR:—At a meeting of the Faculty of the Humboldt Medical College, of this city, held on the 9th inst., the Secretary was requested to notify you of their approbation of, and their acquiescence in, the plan to change the system of Medical College instruction, and their readiness for its early practical adoption. However, they would suggest that in Resolution 2nd, *three* be substituted for *four*, as indicative of the number of years to be required for study; that in Resolution 4th, *medical ethics* be entirely dropped out; and further—they would reserve to themselves the privilege of adding whatever other branches they deemed expedient or necessary. Submitted by yours very respectfully, A. J. STEELE, *Rec. Sec. pro tem.*

A. HAMMER, M.D., *Prof. and Dean of the Faculty.*

---

SAN FRANCISCO, *Oct. 16th, 1867.*

To N. S. DAVIS.

DEAR SIR:—I am instructed by the Trustees and Faculty of the Toland Medical College to acknowledge the receipt of your circular, on the subject of medical education, and to inform you that they most heartily approve of the resolutions set forth, and hereby agree to adopt them, as soon as the same course is pursued by the Colleges named in your circular. Very respectfully and truly yours, THOMAS BENNETT, *Dean.*

---

CHICAGO MED. COLLEGE, CHICAGO, *Oct., 1867.*

To. N. S. DAVIS, S. O. GROSS, F. DONALDSON, GEO. P. BLACKMAN, *Committee.*

MESSRS:—Your circular addressed to the Faculty of the

Chicago Medical College, embodying the action of the Convention of Delegates from Medical Colleges, as been received and presented to the Faculty and Trustees; and I am duly authorized to communicate to you in their behalf the following reply:

The Chicago Medical College, organized in 1859, adopted at the beginning the full *curriculum* recommended by the late Teachers' Convention; instituted thirteen professorships; made the regular annual college term—five calender months; and adopted the progressive method of instruction, by dividing the branches entering into the *curriculum* into two series—the junior and senior—requiring the students attending their first course to so far restrict their attention to the junior series as to bear a full and satisfactory examination in all of them, before they could advance in their next course to the senior series. The success attending this plan, and the manifest advantages to the student have caused the Faculty and Trustees not only to cordially sanction the plan proposed in your circular, but to adopt it, and carry it practically and fully into effect, at the session of 1868-9, whether it is adopted by other medical colleges or not. If they omit anything from the full plan recommended by the Convention, it will be the *fourth* year of study, and the Latin and Greek mentioned in the preliminary requirement. Assuring you that this Faculty will cordially co-operate in any concerted measures for elevating and systematizing the work of educating men for so responsible a profession as that of Medicine. I remain yours truly,

N. S. Davis, *Dean of Faculty of Chicago Med. College.*

---

Washington, *April 28, 1870.*

I am informed by a member of the Faculty of the Medical Department of Williamette University, at Salem, Oregon, that the communication of the Committee was received and acted upon by that Faculty, and a copy of such action sent to the Committee, but the same has not reached me. On his authority, I have included it among the Institutions fully approving the plan proposed by the Convention of 1867.

N. S. Davis.

PHILADELPHIA, *April 11, 1868.*

*Gentlemen:*—It is my duty to inform you that, at a meeting of the Faculty of the Jefferson Medical College, of Philadelphia, held this day, Professors Gross and Rand were appointed Delegates to the next meeting, at Washington.

At the same meeting, it was resolved that whenever a majority of the prominent schools of the country give their assent to the proposed changes in the curriculum, the Jefferson Medical College will, with the permission of the Board of Trustees, heartily co-operate.

I am, gentlemen, with much respect, your obedient, humble servant, ROBLEY DUNGLISON,

*Dean of the Faculty of the Jeff. Med. Coll., of Philadelphia.*

Messrs. N. S. Davis, S. D. Gross, Geo. C. Blackman, F. Donaldson, Committee.

---

UNIVERSITY OF MICHIGAN, *March 20, 1868.*

TO DR. N. S. DAVIS, CHAIRMAN OF COMMITTEE OF THE CONVENTION OF MEDICAL TEACHERS, &c.:

The Medical Faculty of the University of Michigan have received the circular, sent by your Committee to Medical Colleges, and have carefully considered the general plan proposed for revising the system of medical education in this country, and the various propositions in detail, both in reference to their intrinsic merits and their practicability, and have come to the conclusion that the plan referred to is imperfect, and, in some respects, injudicious in its details, and quite impracticable in the present state of the profession among us.

From all the information in our possession, we are of the opinion that its general adoption cannot be secured. These conclusions have not been arrived at through any want of appreciation of the disgraceful condition of medical education among us, or of the importance of medical reform.

It is but just to the Medical Faculty and the Regents of the University, to say that we have long felt the need of such reform, and have, by the institution of preliminary examinations, by the lengthening of the term of instruction to six months, by requiring faithful attendance upon instruction, and giving certificates of the time of such attendance, by requiring of candidates for graduation frequent exercises in writing, in addition to oral examination, and by furnishing the facilities for practical work on a large scale in the chemical and pharmaceutical laboratory, by requiring a large amount of careful dissections, and by rigid final examinations, endeavoring to do what we could toward effecting such reform. But the changes proposed, we regard as too sweeping and extreme; and, even were its main features adopted, the apportionment of the various subjects for the different years is unequal and imperfect.

Without giving the reasons for the same in detail, we propose the following modifications:—

*1st.* Every student applying for matriculation in a Medical College, shall be required to show, either by satisfactory certificate or by direct examination, by a Committee of the Faculty, that he possesses a proper moral and intellectual character; a good English education, including a proper knowledge of the English language, and a respectable acquaintance with its literature and with the art of composition; the more elementary mathematics, including the chief elements of algebra and geometry; the elements of the natural sciences, and such a knowledge of language as will enable him to understand the technical terms of the profession; and the certificate presented, or the result of the examination thus required, shall be regularly filed, together with evidence of the time of commencing medical studies, as a part of the record of each Medical College.

*2d.* Every medical student shall be required to study medicine three and a-half years—deducting, however, six months of time of study in the case of those who may be graduates of a respectable College, requiring four years of the higher studies to complete the course; and, during this time, he must attend two regular courses of Medical College instruction, before being admitted to an examination for the degree of Doctor of Medicine.

*3d.* The minimum duration of a regular lecture term shall be eight months,

24

*4th.* Every Medical College shall embrace in its curriculum the following branches, *viz.:*—Descriptive and Practical Anatomy; Physiology and Histology; Chemistry, inorganic and organic; Materia Medica; Urinalysis and Toxicology; General Therapeutics; Hygiene; Surgical Anatomy; Operations of Surgery; Medical Jurisprudence; Medical Ethics; Practice of Medicine; Practice of Surgery; Obstetrics; Diseases of Women; Diseases of Children; Clinical Medicine and Clinical Surgery; and these several branches shall be divided into two groups, or series, the more elementary subjects, *viz.:*—Descriptive and Practical Anatomy, Physiology and Histology, Chemistry, Materia Medica, Pathological Anatomy, General Pathology and General Therapeutics, being chiefly taught during the first four months of the course, while the more advanced and practical branches, *viz.:*—The Practice of Medicine, Practice of Surgery, Obstetrics, Diseases of Women and Diseases of Children, including Clinical Medicine, Clinical Surgery, Urinalysis and Toxicology, Medical Jurisprudence, Medical Ethics, and Personal and Public Hygiene, shall be chiefly taught during the last four months of the term.

*5th.* Every Medical College shall immediately adopt some effectual method of ascertaining the actual attendance of the student upon its lectures and other exercises, and also, before graduation, of the actual time devoted to the study of medicine out of College; and, at the close of each College session, or of the attendance of the students, a certificate specifying the time and the course of instruction actually attended, shall be given, and such certificate only shall be received by other Colleges, as evidence of such attendance.

This plan contemplates a positive and respectable standard of preliminary education; a longer time of professional study; a systematic and successive order of studies; a certain amount of Clinical instruction, and a *repetition* of the courses of lectures to the student, thereby securing the correction of misapprehension; a more thorough understanding of the various subjects, and a more permanent lodgment of them in the memory.

The present proposed method of accomplishing these objects is, in our judgment, far more simple and easy of execution than the one proposed by the Delegates from Medical Colleges, contained in the circular, and also, in our judgment, it is infinitely more likely to be adopted by the schools. For these reasons, we propose the amendment above stated.

In behalf of Medical Faculty of the University of Michigan.

A. B. PALMER, *Chairman of Committee.*

---

CINCINNATI, *March 9, 1868.*

N. S. DAVIS, M.D., CHAIRMAN COM. ON MED. EDUCATION:

*Sir:*—The Faculty of Miami Medical College respectfully reply to your Committee, that we regard the suggestions contained in your circular as of grave importance, and that they should receive the careful consideration of all the schools.

In *part*, we heartily endorse the action of the Convention, but in part we do not think the present state of medical society in the United States will warrant the attempt to introduce certain of the proposed changes.

Resolution 1st is approved.

Resolution 2d we deem out of the question at present; but are willing to make rule of three years rigid.

Resolution 3d, approved.

Resolution 4th, so far as the curriculum, number of teachers, and clinical requirements are concerned, we heartily indorse them, and carry them out in our school.

That portion of the resolution proposing a general revolution in the *plan* of teaching, *i.e.*, grading, etc., we deem impracticable at this time, even if desirable, *per se*, of which we are not satisfied. We believe it better to leave the *details* of teaching to the judgment of the individual schools.

Resolution 5th, approved.

The reasons for objecting to *four* years' study are, that students are generally too poor, and the community will patronize those who have studied much shorter time. The result would be to throw a large number of students into practice, less perfectly educated than at present.

The same objectious will apply to enforcing the rule of three courses of lectures. If these rules were adopted, the result would be to throw a large proportion of students into schools which would not adopt the regulations, and to lessen, with this class, the standard of requirement, and the facilities afforded them for education.

The result of this would be a great diminution of patronage of all good Colleges, and particularly of Western Colleges that made these requirements, as students would attend institutions not adopting the regulations. The effect would be to keep the latter alive, and to build up new ones of the same class.

We object, therefore, to such vital portions of the plan, that we suppose it scarcely necessary to name any time for inaugurating changes. Respectfully,

WM. H. TAYLOR, *Secretary P. T.*

---

CHARLESTON, *February 25, 1868.*

TO DR.'S N. S. DAVIS, S. D. GROSS, G. C. BLACKMAN, AND F. DONALDSON, COMMITTEE APPOINTED BY CONVENTION OF TEACHERS OF MEDICAL COLLEGES:

*Gentlemen:*—The circular addressed by you, under the authority of the Convention of Teachers of Medical Colleges, to the Faculty of the Medical College of the State of South Carolina, has been received, and, in compliance with your request, the following answer is respectfully tendered:—

The entire subject matter involves several propositions:—

*1st.* Expediency and practicability.

*2d.* The general and special details of the plans proposed.

*3d.* Suggestions designed to improve those plans.

Upon each of these heads, the Faculty beg leave, respectfully, by way of answer, to offer a few remarks.

The expediency and importance of the measures and objects proposed are too patent to require any argument; and, while with many of the leading Colleges of the Union, the entire practicability is at least probable, it is greatly to be apprehended that, in the Southern States, broken down and impoverished, as they are, with all their resources gone, a policy, otherwise in the highest degree desirable, could not, under existing circumstances, be successfully carried out. Even under more propitious conditions, there is reason to fear that mere voluntary concurrence would fail to secure the important ends proposed. There are so many rival schools—all self-sustaining—that, under the spirit of competition for numbers, an easy morality, unbridled by restraint, would speedily lead to a neutralization of pledges, however sincerely made in the beginning. It is believed that the objections thus anticipated, can only be met by concurrent State legislation. If all of the States could be induced to adopt a uniform system of medical education, taking as their guide the plan proposed by the Convention, the difficulties would be effectually obviated. The General Government, in consequence of its peculiar organization, does not possess the power.

*2dly.* The several propositions embodied by the Convention so fully cover the important questions involved, that they present a strong claim to general sanction, and demand but few suggestions, by way of improvement. But as propositions are invited by the Committee, the following are respectfully offered:—

SEC. 1. In connection with the evidences of preliminary education, would it not be better to require the applicant to construe a plain and easy sentence in Latin and Greek, than to have the requirement so indefinitely expressed?

SEC. 2. It is respectfully suggested, in reference to the four years' study proposed by the Convention, three of which are required to be employed in attendance upon lectures in a College, that the first of these four years employed, as it must be in the wasteful expenditure of time in an office, without instruction or proper guidance, can be of no avail to the pupil. If four years are to be required, would it not be more advantageous to exact an attendance on collegiate or university instruction during the four terms? In all European institutions which have been modeled on the results of long and mature experience, it has been found expedient to divide the scholastic year into two terms or semesters; the first extending from the middle of October or the 1st of November, to the 1st of March; the second, commencing on the 1st of April, terminates on the 1st of August, thus leaving one month in the spring, and two and a half months in autumn, for relaxation from study, and allowing a reasonable time for recreation. This arrangement, it is believed, presents many advantages, not the least of which is, that inasmuch as many of the subjects comprised in the course can be as well taught in the spring and summer, as in winter, others being more advantageously prosecuted during the latter season. And, in addition to this, there is another advantage, *viz.:* the courses, under this distribution, may be so arranged that those which call for the widest range of discussion, as practical medicine, surgery, &c., may be extended through the two semesters, while others of limited extent may be completed in one. This would not only prevent crowding—a great evil under the present system—but allow ample time, during the summer semester, for the advantageous introduction of various collateral subjects, more or less special, but all important, as ophthalmology, ear diseases, dermatology, laryngoscopy, microscopy, chemical analyses, &c.

It is therefore respectfully suggested that instead of a single term of six months, as proposed by the Convention, the scholastic year be divided into two; one for the winter, the other for the summer, as described above, each comprising four months; and that the subjects to be taught be appropriatelty distributed in accordance with this division of time.

Sec. 3. But slight suggestions are proposed in reference to this section. It is nevertheless questionable whether the subjects enumerated could be efficiently taught by a corps of nine professors. Thus, clinical medicine and clinical surgery would perhaps require incumbents, separate from and independent of those engaged in the delivery of didactic discourses on these subjects. As regards the arrangement and grouping of the subjects, a slight modification of the plan proposed by the Contion might perhaps be advantageous. Thus, it is respectfully suggested to separate inorganic and organic chemistry from materia medica, and associate with the two former medical physics—perhaps pharmacy; to add general therapeutics to materia medica, and to associate toxology with medical jurisprudence, to which, in many respects, it legitimately belongs. Ethics may be rather regarded as a collateral study.

The Faculty deferentially, but with great sincerity, offer these remarks; and while they deem it inexpedient, in view of the present relation of their College, to pledge themselves to an adoption of the measures proposed, they take great pleasure in expressing their cordial approbation of the efforts thus made to improve our system of medical education, and thereby rescue it from the reproaches to which it has been so long amenable. With much respect, your obedient servant,

J. J. CHISHOLM, M.D., *Dean of the Faculty.*

It will be seen that the first of these replies is entirely non-committal, while of the remaining nine, four suggest amendments or alterations in the plan proposed, and five unequivocally sanction the plan as a whole, and express a willingness to carry it into practical effect, whenever the other colleges throughout the country will do the same. In addition to these official replies, the Chairman of your Committee has received several letters from individual members of college faculties, and from some of the most eminent in the profession. But as they express only individual opinions, it has not been deemed proper to include any of them in this report.

Your Committee, having been unable to obtain even an expression of opinion concerning the plan proposed by the Convention of 1867, from the faculties of a large majority of the Medical Colleges, have, in accordance with the spirit of their instructions, sent copies of the following circular to all the regular Colleges in the United States.

"The undersigned Committee, in accordance with the instructions of the Convention of Delegates from Medical Colleges, held in Cincinnati, in May, 1867, respectfully and earnestly invite you to send Delegates to a Convention to be held in the City of Washington, on the Friday preceding the first Tuesday in May, 1870; for the purpose of considering all subjects connected with medical college education, and procuring the co-operation of the Schools in carrying out a uniform system of medical instruction. It is very desirable that every regular Medical College in the country should be represented in the Convention.

N. S. Davis,<br>

S. D. Gross,<br>

Geo. C. Blackman,<br>

F. Donaldson,<br>

} *Committee.*

Chicago, Ill., *Dec. 22d, 1869.*

It is in response to this invitation that you are assembled here to-day. It is no part of our object, in presenting this report, to enter upon the discussion of the merits of any proposed plans of improvement, but simply to lay before you the record of what was done in the previous Convention, at Cincinnati, and of what has resulted in relation thereto, since. We cannot willingly close this report, however, without a few words of warning or exhortation. From all our correspondence and intercourse with the profession at large, we are satisfied that if this Convention shall result in the actual adoption, by the Colleges, of the plan proposed at Cincinnati, or something equivalent thereto, and the same shall be carried into effect by the Schools located in the cities, named in the circular of the Committee dated Aug. 1st, 1867, they will be fully and promptly sustained by the whole profession, even to the complete repudiation of the diplomas of all colleges that might attempt to maintain any lower standard of action. But if the Convention shall fail in the accomplishment of any important practical results, it will give a powerful impetus to a long-existing, but partially latent, disposition on the part of the profession to practically nullify the value of all medical college diplomas, by establishing independent Boards of Censors in each State, and refusing to acknowledge as regular members of the profession, or admit to membership in any medical society, local, state, or national, all who do not submit to an examination, and obtain a license from such Board. This will be claimed as the only method by which students of medicine can be made to resort to such colleges as will give them the most complete and thorough courses of instruction in every department of medical knowledge, instead of such as will give them a diploma in the shortest time, and with the smallest expenditure of money, for the sake of increasing the number of their classes. The time has fully come when some important practical results must be obtained.

Let us bring to the work calmness, deliberation, liberality of sentiment, and an honorabie confidence in the abiding integrity of each other, and the work will be accomplished. Individual interests and prejudices are limited and temporary. But the interests, the honor, and the usefulness of a profession, whose existence constitutes an integral part of civilized society; and whose duties are interwoven with the highest and tenderest interests of the human race, must remain to the end of time, In all our deliberations during the continuance of this Convention, let us forget the first, while, with earnest purpose, we keep the latter constantly in view. All of which is respectfully submitted. N. S. DAVIS.

WASHINGTON, D.C., *April 28th, 1870.*

On motion, the propositions in relation to medical education, adopted by the Convention of 1867, were made the basis for the further action of this Convention; and the Secretary placed in the hands of each member a printed copy of the same. The Convention then adjourned until 10 o'clock A.M., to-morrow.

---

SATURDAY — SECOND SESSION.

Meeting called to order by the President, at 10 o'clock A.M. The Secretary read the minutes of the previous meeting, and they were approved. A recess of ten minutes was taken, to allow the Committee on Credentials to register new members.

When the Convention was again called to order, Prof. S. M. Bemis, Chairman of Committee on Credentials, reported the following additional members:

Kansas City Medical College. { Prof. John M. Forrest. / " A. P. Lankford.

Missouri Medical College.—Prof. A. Hammer.

Med. Depart. University, Nashville. { Prof. W. K. Bowling, / " W. G. Briggs.

National Med. College, D.C. { Prof. W. P. Johnston. / " N. S. Lincoln.

Med. Depart. University, Maryland.—Prof. F. Donaldson.

Prof. N. S. Davis offered the following resolutions, for the purpose of facilitating the transaction of business, and they were unanimously adopted:

*Resolved*, That all motions and resolutions except to adjourn, lay on the table, or postpone, must be in writing, with the name of the author attached.

*Resolved*, That the name of every member who may be recognized as occupying the floor, must be distinctly announced by the President, before he is allowed to proceed.

*Resolved*, That no member shall be permitted to speak more than once on the same subject, until all others have spoken who desire to do so.

The Secretary announced the reception of a dispatch, saying that John A. Murphy, of the Miami Medical College, and Theophilus Parvin, of the University of Louisville, were detained at Grafton, Va., by failure to make connection in the railroad trains.

Prof. N. S. Davis offered the following resolution, which was adopted:—

*Resolved*, That the several propositions adopted by the Convention at Cincinnati, in 1867, be taken up separately in the order they stand in the printed report.

The first proposition, which related to the standard of preliminary education, was read by the Secretary. After some discussion, concerning the question whether the action which might be taken was to be considered binding on the institutions represented, it was distinctly claimed by the delegates from Philadelphia and several other places, that they had no authority to pledge their respective schools until their action should have been submitted to their respective faculties. Prof. J. S. Moore offered the following amendment:—

Strike out of the proposition all between the word "education" and the words "and the certificate," and inserting "with a sufficient knowledge of the construction of the language to enable him to understand medical technicalities."

After remarks by Prof. J. S. Moore and Prof. D. W. Yandell, in favor of the motion, Prof. A. Hammer offered the following amendment to the amendment of Prof. Moore :—

To strike out of the original proposition all that related to Greek and Latin, and retain the rest. He protested against the views expressed by Professors Moore and Yandell, and advocated a fair standard of education, but was willing to omit the ancient languages, if it would be the means of making the rest of the standard more likely to be adopted and carried into practical effect.

Profs. Reyburn, Loomis, and Cox, of Washington, advocated the retention of the original proposition, without amendment.

Prof. D. W. Yandell replied, in substance, that all resolutions and recommendations on the subject were of no avail. The community would employ who they pleased, as doctors, whether they had any education or not; and medical students would go to any College they pleased, if they had money enough to pay the fees, entirely regardless of any standard of preliminary requirements.

Prof. N. S. Davis discussed the subject fully, claiming that the positions occupied by Prof. Yandell, and others on the same side, were wholly untenable. The reason why so large a number of medical students presented themselves to the Colleges, totally without any adequate general education, was simply because the schools required none. Suppose all the Literary Colleges and Universities in the country should open their doors to any who might come, without any examination or standard of previous acquirements, how long before they would be filled with students who had not acquired the first elements of education? If the Medical Colleges make a standard, and provide for its practical maintenance, instead of cutting off the supply of students, it would simply cause such young men as desired to study medicine, to first make the necessary preliminary preparation. And not only this, but the example of the Colleges would be followed by all our social organizations, whether local, State, or National, forbidding any of their members from receiving a student into their offices, without the required amount of mental training.

He thought the subject one of paramount importance; in fact, the very foundation on which all other improvements must rest. He would not be tenacious about Greek and Latin. But the responsibility for the present state of things was directly with the schools. It was narrowed to the simple question, whether the College Faculties will honestly unite on what all agree to be desirable and right, and carry it into practical execution, or whether they will let their mutual jealousies and distrust of each other, keep the education of the whole profession in its present condition?

Prof. A. Stille claimed that the action of the American Medical Association, and that of the previous Convention of College Delegates, had had a good effect. They had advanced and moulded public sentiment in the profession, and much progress had actually been made by the schools. He thought further advances must be made in the same way. He therefore moved the following, as a substitute for both the amendments now before the Convention:—

*Resolved*, That, in the judgment of this Convention, the propositions adopted in 1867, by the Convention of Delegates from Medical Colleges, embody a system of collegiate medical education in the highest degree commendable, and which, if they could be generally carried into effect, would tend to elevate the medical profession,—that, nevertheless, the requirements for the degree of Doctor of Medicine must be practically determined by each Medical College for itself, by the average attainments of its students, and by other considerations, of which it alone can judge; and that, consequently, while abstaining from all attempts at dictation, this Convention reiterates, in the strongest manner, its desire that the several Medical Colleges will, in the changes from time to time made by them in the curriculum of study, endeavor to conform them to the general plan which was recommended by the Convention of 1867, and adopted in the same year by the American Medical Association.

Prof. Samuel Logan thought the substitute out of order, inasmuch as it carried with it all the subsequent propositions, as well as the one directly under discussion.

The President decided the substitute to be in order.

Its adoption was opposed by Professors Hammer, Logan, J. B. Johnson, and Bemiss, who claimed that they had come to the Convention with such instructions from their respective schools; that they were prepared to act on all the propositions, and with binding effect.

Prof. S. D. Gross, having called the Vice-President to the chair, took the floor, and stated that the whole question was in a nut-shell, and a very small shell at that. It was simply, "Will the American Medical Colleges combine, and go forward to a proper standard of education, or not?" Will the Colleges continue to stultify themselves with resolutions, or will they act? He thought the schools alone responsible, and claimed that they could accomplish the work in an hour, if they would. He said it could be done by no other power—the American Medical Association being powerless in the matter.

In taking the vote on the substitute offered by Prof. Stille, a division of the house was called for, and resulted in *five* affirmative votes, and *fifteen* in the negative. The substitute was rejected.

The question recurring on the amendment to the amendment, as offered by Prof. Hammer, it was opposed by Prof. Gross, and, with the consent of the Convention, it was withdrawn.

The question recurring on the amendment offered by Prof. Moore, Prof. Forrest again raised the question, whether the action the Convention may take is to be regarded as final and obligatory upon the Colleges represented, or only advisory?

A conversational discussion ensued, which developed the fact that a majority of the representatives from Colleges in the West and Southwest had come with definite instructions in favor of advancement and final action; while the representatives from both the schools in Philadelphia, and several others, disclaimed having any instructions, and that, whatever might be done, would have to be submitted to the Faculties of the schools for ratification.

In answer to an inquiry about the terms of the call for this Convention, Prof. Davis explained that the results of the previous Convention, consisting of the propositions now before this body, were laid before the Faculties of all the Colleges, known to exist in the country, more than two years since, and it was certainly hoped and expected, that, in sending delegates to this Convention, they would so instruct them as to permit final action; but if such had not been the case, we had now no alternative but to regard whatever action we take as advisory.

Prof. Gross moved a reconsideration of the vote on the substitute offered by Prof. Stille, which was seconded. The reconsideration was opposed by Professors Logan and Moore.

Prof. Davis wished it to be understood that most of the Colleges in the West and South were ready to act, and declared that if the old institutions in Boston, New York and Philadelphia, would lay aside their petty rivalries, and act on what they all acknowledged to be right, every College in the Mississippi Valley and beyond it, would cordially go with them. He wished the responsibility to rest where it justly belonged. If, however, we could only advise, he was in favor of holding what we had got at present, and trust to time and Providence for the future. It would be found that there was more than one road to the object sought.

The vote on the reconsideration was carried in the affirmative.

Prof. Gross moved the adoption of Prof. Stille's substitute, which was carried by a large majority.

Prof. Samuel Logan then offered the following resolutions:

*Whereas*, This Convention has failed to secure the assent of a majority of the regular Medical Colleges of the United States, to the system of improvement in medical education, recommended at its last session; and,

*Whereas*, It is the opinion of this Convention that the best means by which a judicious system of gradual improvement in medical education can be inaugurated by the Medical Colleges of this country, will be found in the *associated action* of such Colleges as will unite for that purpose; it is hereby

*Resolved, 1st.* That a committee of nine be appointed, whose duty it shall be to communicate with the Faculties of all the regular Medical Colleges in the United States, with the view to ascertain how many, and which may be willing to become members of an *Association of Medical Colleges*, having for its prime object the improvement of medical education.

*2d.* That the Chairman of said Committee be instructed, as soon as he shall have received affirmative replies from ten (?) regular Colleges, to inform each Faculty so consenting, of the fact, and to request that each Faculty elect one or more delegates, to convene on the Friday before the day appointed for the meeting of the American Medical Association, in 1871, and at the place of meeting chosen by that body—said delegates to be fully authorized to pledge their respective Faculties to whatever definite plan of improvement in medical education may be adopted by the body in Convention.

*3d.* That it is hereby recommended that said delegates organize themselves, in behalf of their respective institutions, into a permanent *Association of Medical Colleges* for the above-mentioned object, and with the view of co-operating with the American Medical Association and the profession at large, in efforts to accomplish so desirable an end.

*4th.* That Prof. N. S. Davis, the Chairman of the Committee, appointed by the Convention of 1867, to communicate with the Medical Colleges on the same subject, be made the Chairman of this Committee, and that the Committee be authorized to fill any vacancies which may occur in its ranks.

After the reading of the resolutions, Prof. Lankford moved that the Convention adjourn to 8 o'clock P.M., for their further consideration, which motion was seconded and carried.

EVENING SESSION.

The Convention was called to order at 8 P.M.—Prof. Yandell, Vice-President, in the Chair.

The following additional delegates presented their credentials, and took their seats:—

| | |
|---|---|
| University of Louisville, | Prof. Theopholis Parvin. |
| Miami Medical College, | " J. A. Murphy. |
| Medical College of Alabama, (Mobile), | " Edward P. Gaines. |
| University of Buffalo, | " Chas. A. Lee. |

The Secretary read the resolutions offered by Prof. Logan, previous to adjournment.

On motion of Prof. Bemiss, they were unanimously adopted.

The Vice President in the Chair, filled the Committee called for in the first resolution, as follows:—

Prof. N. S. Davis, of Chicago, Chairman.
" Samuel Logan, of New Orleans.
" A. Hammer, of St. Louis.
" T. Parvin, of Louisville.
" S. D. Gross, of Philadelphia.
" A. C. Post, of New York.
" Geo. C. Shattuck, of Boston.
" Geo. C. Blackman, of Cincinnati.
" A. P. Talley, of Charleston.

On motion of Prof. Logan, it was resolved that a complete copy of the proceedings of this Convention be transmitted by the Secretary to the Secretary of the American Medical Association; another copy to the Committee of Nine, just appointed; and another to the medical periodicals of the country.

Prof. A. Hammer offered the following resolution, seconded by Prof. Moore:—

*Resolved*, That the attempt, on the part of any existing schools, to reduce their tuition fees, or of any new College to be started, to underbid its neighbors, shall be by this body considered and pronounced to be an act of dishonesty.

After some discussion, participated in by Professors Hammer, Murphy, and Davis, the mover, with the consent of the house, withdrew the resolution.

On motion of Prof. Hammer, the thanks of the Convention were tendered to the officers, for the able manner in which they had discharged their duties; and to the Faculty of Georgetown Medical College, for the use of their building.

On motion of Prof. Bemiss, the Convention adjourned *sine die.*

N. S. DAVIS,
*Secretary of Convention.*

